# Novel Tools for Single Comparative and Unified Evaluation of Qualitative and Quantitative Bioassays: SS/PV–ROC and SS-J/PV-PSI Index–ROC Curves with Integrated Concentration Distributions, and SS-J/PV-PSI Index Cut-Off Diagrams

**DOI:** 10.3390/diagnostics14090951

**Published:** 2024-04-30

**Authors:** Peter Oehr

**Affiliations:** Faculty of Medicine, University of Bonn, 53113 Bonn, Germany; prof.oehr@gmx.de

**Keywords:** urinary bladder cancer, diagnosis, UBC^®^ Rapid biomarker, ROC curve, SS–ROC curve, PV–ROC curve, SS-J index, PV-PSI index, PV-PSI index–ROC curve, index–ROC curve, SS-J/PV-PSI index cut-off diagram, reciprocal SS-J index

## Abstract

**Background:** This investigation is both a study of potential non-invasive diagnostic approaches for the bladder cancer biomarker UBC^®^ Rapid test and a study including novel comparative methods for bioassay evaluation and comparison that uses bladder cancer as a useful example. The objective of the paper is not to investigate specific data. It is used only for demonstration, partially to compare ROC methodologies and also to show how both sensitivity/specificity and predictive values can be used in clinical diagnostics and decision making. This study includes ROC curves with integrated cut-off distribution curves for a comparison of sensitivity/specificity (SS) and positive/negative predictive values (PPV/NPV or PV), as well as SS-J index/PV-PSI index–ROC curves and SS-J/PV-PSI index cut-off diagrams (J = Youden, PSI = Predictive Summary Index) for the unified direct comparison of SS-J/PV results achieved via quantitative and/or qualitative bioassays and an identification of optimal separate or unified index cut-off points. **Patients and Methods:** According to the routine diagnostics, there were 91 patients with confirmed bladder cancer and 1152 patients with no evidence of bladder cancer, leading to a prevalence value of 0.073. This study performed a quantitative investigation of used-up test cassettes from the visual UBC^®^ Rapid qualitative point-of-care assay, which had already been applied in routine diagnostics. Using a photometric reader, quantitative data could also be obtained from the test line of the used cassettes. Interrelations between SS and PV values were evaluated using cumulative distribution analysis (CAD), SS/PV–ROC curves, SS-J/PV-PSI index–ROC curves, and the SS-J/PV-PSI index cut-off diagram. The maximum unified SS-J/PV-PSI index value and its corresponding cut-off value were determined and calculated with the SS-J/PV-PSI index cut-off diagram. **Results:** The use of SS/PV–ROC curves with integrated cut-off concentration distribution curves provides improved diagnostic information compared to “traditional” ROC curves. The threshold distributions integrated as curves into SS/PV–ROC curves and SS-J/PV-PSI index–ROC curves run in opposite directions. In contrast to the SS–ROC curves, the PV–ROC and the novel PV-PSI index–ROC curves had neither an area under the curve (AUC) nor a range from 0% to 100%. The cut-off level of the qualitative assay was 7.5 µg/L, with a sensitivity of 65.9% and a specificity of 63.3%, and the PPV was 12.4% and the NPV was 95.9%, at a threshold value of 12.5 µg/L. Based on these set concentrations, the reader-based evaluation revealed a graphically estimated 5% increase in sensitivity and a 13% increase in specificity, as compared to the visual qualitative POC test. In the case of predictive values, there was a gain of 8% for PPV and 10% for NPV. The index values and cut-offs were as follows: visual SS-J index, 0.328 and 35 µg/L; visual PV-PSI index, 0.083 and 5.4 µg/L; maximal reader Youden index, 0.0558 and 250 µg/L; and maximal PV-PSI index, 0.459 and 250 µg/L, respectively. The maximum unified SS-J/PV-PSI index value was 0.32, and the cut-off was 43 µg/L. The reciprocal SS-J index correctly detected one out of three patients, while the reciprocal PV-PSI index gave one out of twelve patients a correct diagnosis. **Conclusions:** ROC curves including cut-off distribution curves supplement the information lost in “traditionally plotted” ROC curves. The novel sets of ROC and index–ROC curves and the new SS/PV index cut-off diagrams enable the simultaneous comparison of sensitivity/specificity and predictive value profiles of diagnostic tools and the identification of optimal cut-off values at maximal index values, even in a unifying SS/PV approach. Because the curves within an SS-J/PV-PSI index cut-off diagram are distributed over the complete cut-off range of a quantitative assay, this field is open for special clinical considerations, with the need to vary the mentioned clinical diagnostic parameters. Complete or partial areas over the x-axis (AOX) can be calculated for summarized quantitative or qualitative effectivity evaluations with respect to single and/or unified SS-J and PV-PSI indices and with respect to single, several, or several unified assays. The SS-J/PV-PSI index-AOX approach is a new tool providing additional joint clinical information, and the reciprocal SS-J indices can predict the number of patients with a correct diagnosis and the number of persons who need to be examined in order to correctly predict a diagnosis of the disease. These methods could be used in applications like medical or plant epidemiology, machine learning algorithms, and neural networks.

## 1. Introduction

In clinical patient care, the requirements for an accurate interpretation of diagnostic data are different: patients present with unclear or specific symptoms that may be associated with (early) cancer or another benign disease [1,2,3], and the prevalence of cancer among all subjects of interest is quite different in epidemiologic cancer screening studies or on an outpatient ward of an oncological clinic [1,2,3].

Laboratory assays for blood-based tumor markers often show a considerable overlap of value distributions of cancer and non-cancer cohorts, requiring a tradeoff between sensitivity and specificity when defining an appropriate cut-off [1,2,3]. To directly compare different diagnostic tools, so-called receiver operating characteristic (ROC) curves have been used for many years, providing information about the sensitivity/specificity (SS) profiles over the whole range of possible cut-offs [2,3,4,5,6].

Traditional ROC curves, only used for the investigation of SS, without integrated threshold distribution curves, were first developed during World War II to assess the ability of radar operators to differentiate signals (e.g., enemy aircraft) from noise (e.g., a flock of birds), and later for signal theory [7,8,9]. Their potential for medical diagnostics was first observed in radiology by Lusted in 1960 [10]; however, this was only after the publication of the text by Swets and Pickett [11]. Its expansion to other medical fields was first extensively used in radiology to evaluate medical imaging devices [12]. Since that time, SS–ROC curves have widely been adapted as standard tools in diagnostic medicine studies [13,14,15,16,17].

ROC curves can also be used in plant epidemiology [18,19], and can be applied by including the area under the ROC curve in the evaluation of machine learning algorithms [20] or to improve the accuracy, scalability, and performance of graph neural networks [21].

The first SS–ROC curves for clinical biomarker diagnosis were introduced in 1981 [22]. They included SS–ROC curves for the diagnostic analysis of in vitro bioassays (CEA, TPA, AFP, TAG, and beta-HCG) in patients with tumor diseases (lung, base, and testicular carcinomas in comparison to control groups with/without benign diseases). This article can be obtained on the Internet (Oehr Derigs Altmann ResearchGate).

Using SS–ROC curves, biomarkers can be compared using the area under the curve (AUC), using sensitivities at defined specificities that are clinically required—such as 95% specificity versus benign control groups—or using optimized cut-offs that provide a high sensitivity at a high specificity [3,15,17,18].

Sensitivity and specificity indicate the concordance of a test with respect to a chosen referent, while PPV and NPV, respectively, indicate the likelihood that a test can successfully identify whether people do or do not have a target condition, based on their test results.

In contrast to the SS–ROC curve, which is independent of disease prevalence, the geometric characteristics of the predictive value (PV)–ROC curve vary with prevalence [23,24]. Shiu and Gatsonis were the first to present methods for the joint study of the positive (PPVs) and negative predictive values (NPVs) of diagnostic tests. They defined the predictive receiver operating characteristic as a PROC curve that consists of all possible pairs of PPVs and NPVs as the threshold for test positivity variations [23].

The first works presenting a unifying approach to the description of performance measures for both types of curves were made available in a Special Issue of Entropy [18]. This Special Issue showcases the current approaches to epidemiological applications of information theory and represents the first wide-ranging overview of epidemiological applications since the first publication of applications of information theory to epidemiology referring to plant pathology [20]. The 2020 Issue represents the first wide-ranging overview of epidemiological applications, together with ten research papers, five of which were contributed by authors whose primary interests are in phytopathological epidemiology and five by authors primarily interested in clinical epidemiology. These articles contribute to an improved understanding of the ways that ROC and PROC curves can jointly contribute to the analysis of diagnostic information [18].

It took nearly 40 years until the first empirical biomarker PV–ROC curve appeared in the literature. According to the present state of affairs, this seems to be the only PV–ROC curve publication on biomarkers so far [25].

In this manuscript, data from urinary bladder cancer and from patients without evidence of bladder cancer are investigated using the UBC^®^ Rapid test for visual qualitative analysis and the UBC^®^ Rapid Concile^®^ Ω100 photometric reader for quantitative determination.

The data are used for demonstration, partially to compare ROC methodologies and also to show how both sensitivity/specificity and predictive values can be used in clinical diagnostics and decision making.

One of the goals of this study was to directly evaluate and compare quantitative and qualitative bioassays according to SS and PV values. Another goal was to show how SS and PV results differ in their clinical applicability and to explore whether a transparent method can be proposed to identify appropriate cut-offs for both sensitivity/specificity and predictivity at a given cut-off level. The results of such evaluations naturally include conclusions about the applicability of the assays.

Concentration distribution curves were integrated into the ROC curves to enable the direct comparability of SS– and PV–ROC curves and to optimize the information content of ROC curves.

The analysis was carried out by including SS/PV–ROC curves (synonym: SS/PV–ROC plots) as well as newly developed SS-J/PV-PSI index–ROC curves and a novel (SS-J/PV-PSI) index cut-off diagram (J = Youden, PSI = Predictive Summary Index). These enabled the comparison of the sensitivity/specificity and predictive profiles of diagnostic tools in the dependency of disease prevalence and the identification of optimal cut-off values in a unifying approach. This is a novel information–theoretical application, which might be of interest to epidemiologists and diagnosticians in both medicine and plant pathology.

## 2. Methods

### 2.1. Cumulative Distribution Analysis (CDA)

According to Kallner, although the ROC curve conveys a substantial amount of information, some is lost in the process, most importantly the value of the cut-off. For this reason, an ROC will not directly depict the relationship between the cut-off and the performance, and it may be important to offer a tool to infer the effect of modifying the cut-off value on the sensitivity and specificity. The loss of information in the ROC curve can be compensated for by using a cumulative data analysis (CDA) plot, which displays the sensitivity and specificity on the Y-axis against the cut-off values on the X-axis. This allows for the graphical depiction of the real-time effects of changing the cut-off value and complements and supports in illustrating and deciding on the characteristics of a test [26]. Using the Microsoft software Office Excel 97-2004, graphs were created by clicking on the Insert tab and then clicking on Point Diagram (X, Y).

### 2.2. SS– and PV–ROC Curves Including Cut-Off Value Distribution Curves

Concerning ROC curves, a “true negative” is the event in which the test makes a negative prediction and the subject has a negative result under the gold standard, and a “false negative” is the event in which the test makes a negative prediction and the subject has a positive result under the gold standard. 

The curves were constructed after thresholds or cut-off levels were introduced to determine true positive/negative and false positive/negative values corresponding to each threshold. This was undertaken at set concentrations determined by the author, including 5, 7.5, 10, 30, 50, 90, 110, 250, and 300 µg/L. The results for true positive/negative and false positive/negative values were used to calculate sensitivity, specificity, and predictive values in order to establish the SS/PV–ROC curves, SS-J/PV-PSI index–ROC curves, and the SS-J/PV-PSI index cut-off diagrams, including the detection of optimal cut-off values.

Data for sensitivity/specificity pairs, as well as positive/negative predictive values, were created on the basis of the varied threshold values for the whole patient cohort, as well as for the benign disease group.

Based on these values, the related values for sensitivity and specificity, as well as the positive and negative predictive values (PPVs and NPVs), could be calculated. Using the Microsoft software Office Excel 97-2004, graphs for the ROC curves were created including cut-off distribution curves by clicking on the Insert tab and then clicking on Point Diagram (X, Y).

In an SS–ROC curve, values for sensitivity are plotted on the Y-axis and those for 1-specificity (for visual purposes) are plotted on the X-axis, whereas in a PV–ROC curve, the PPVs are plotted on the Y-axis and those for 1-NPVs are plotted on the X-axis instead.

### 2.3. SS Index–ROC and PV Index–ROC Curves

Several indices are available to summarize the information in an ROC graph. In order to find optimal cut-offs for the comparison of single or unified evaluations, index–ROC curves were developed using the index suggested by W. J. Youden in 1950 for SS–ROC curves [24] and the Predictive Summary Index (PSI) developed by Linn and Grunau in 2004 for PV–ROC curves [27].

The Youden index (J) is a method applied for the evaluation of optimal cut-off values in SS–ROC curves to select a maximum level of sensitivity/specificity [28]. It is defined as J = sensitivity + specificity − 1. This index can take any value between −1 (sensitivity or specificity = 0) and +1 (sensitivity = specificity). The maximum value of the Youden index is 1 (perfect test) and the minimum value is 0 when the test has no diagnostic value. The minimum occurs when sensitivity = 1 − specificity, which is represented by an equal line (diagonal) in the ROC diagram. The reciprocal of J is interpreted as the number of persons with a given disease who need to be examined in order to correctly detect one person with the disease [27].

The PV index (PSI) is a method applied for the evaluation of optimal cut-off values in PV–ROC curves to select a maximum level of predictive values. It shows that the overall gain in certainty can be expressed in the form of the following expression: PSI = PPV + NPV − 1. 

The maximum value of the PSI index is 1 (perfect test), which indicates that the test is always correct and there are no false negatives, while with a test that has no predictive value, PSI = 0. In the case of a PV–ROC curve diagram, the line of the diagonal does not represent the minimum, because PV–ROC curves can cut the diagonal [23,24,25]. The reciprocal of PSI is suggested as the number of persons who need to be examined in order to correctly predict a diagnosis of the disease [27].

For reasons of systematic terminology (nomenclature) in this and future manuscripts, including further novel developed types of ROC curves by the author of this manuscript, and in order to differentiate the Youden index from other novel ROC-curve-connected indices, like those for predictive values (PVs), the terminology SS-J index is used for the index introduced by Youden [28], and PV-PSI index is used for the index proposed by Linn and Grunau [27].

### 2.4. SS-J Index/PV-PSI Index–ROC Curves

Accordingly, the SS-J index/PV-PSI index–ROC curves were constructed, after thresholds or cut-off levels were introduced to determine true positive/negative and false positive/negative values corresponding to each threshold. Using the Microsoft software Office Excel 97-2004, graphs for the ROC curves were created including cut-off distribution curves by clicking on the Insert tab and then clicking on Point Diagram (X, Y). In the SS-J index–ROC curves, the resulting index values for sensitivity are plotted on the Y-axis, and those for 1 − specificity (for visual purposes) are plotted on the X-axis, whereas in the PV-PSI index–ROC curves, the PPV index values are plotted on the Y-axis and those for 1-NPV are plotted on the X-axis. SS–ROC curves have an area under the curve, and PV–ROC curves do not. SS-J index- and PV-PSI index–ROC curves can cross the diagonal of a graph, and both can be compared in a single graph at varied cut-off levels.

### 2.5. Quantitative Determination of Visual POC Results

A quantitative determination of visual POC results was derived graphically either from CAD or within the ROC curves by comparing the SS or PV results received from the concentration-dependent color reaction on the test line (used as a cut-off value containing a predefined concentration) of the POC device with those of the qualitative visual POC test.

### 2.6. SS-J/PV-PSI Index Cut-Off Diagram

The cut-off levels within the PV–ROC curve as well as the PV index–ROC curve diagrams run opposite to those of the SS-derived curves, and the PV-derived curve distributions change their position in relation to both the X- and Y-axes, with changing prevalence and cut-off level values [23,24]. Under these conditions, a direct comparison to SS-J and PV-PSI index levels is difficult. In the novel developed (SS-J/PV-PSI) index cut-off diagrams, cut-off values were plotted on the X-axis, whereas both SS-J and PV-PSI index values at each selected SS and PV cut-off were plotted against the Y-axis. At this level, any SS-J index value can be directly related to its corresponding PV-PSI index value at varied cut-off levels within a single diagram. Using the Microsoft software Office Excel 97-2004, graphs were created by clicking on the Insert tab and then clicking on Point Diagram (X, Y).

### 2.7. Reciprocal SS-J/PV-PSI Index Cut-Off Diagram

Using the same method, the reciprocal SS-J/PV-PSI index cut-off diagrams were plotted in order to indicate the number of persons with a given disease who need to be examined in order to correctly detect one person with the disease, and the reciprocal of PSI was used to indicate the number of persons who need to be examined in order to correctly predict a diagnosis of the disease at varied cut-off thresholds.

### 2.8. Patient Data and Methods

In order to show the applicability of the different ROC curves, data from a clinical tumor marker study were used. This investigation used data originating from former market research in 2017, made in order to test the usability of a newly introduced reader system for point-of-care assays. This study was a quantitative investigation of used-up test cassettes from the visual UBC^®^ Rapid qualitative point-of-care assay which had already been applied in routine diagnostics. Using a photometric reader, quantitative data were obtained from the test line of the used cassettes. This study involved a quantitative evaluation using leftover consumables that are normally thrown in the trash, and does not describe research on humans or animals. That is why Ethics Committee or Institutional Review Board approval was not required for this manuscript.

This study included only traditional SS–ROC curves without inserted cut-off distribution curves. The aim of this study was to find out whether the quantitative use of the UBC^®^ Rapid test improves the clinical test performance of visual nonquantitative POC testing in the daily routine of common medical practice facilities.

Urinary samples from urological practice facilities in Germany, including 1243 patients suspected of having urinary bladder cancer, were investigated. Prior to sampling, the written consent of the patients for this study was obtained. According to data from the routine diagnostics, 91 patients were confirmed to have bladder cancer and 1152 patients displayed no evidence of bladder cancer, leading to a prevalence value of 0.073.

The exclusion criteria were infections of the urinary tract, invasive treatment, instillations like BCG, pregnancy, and diabetes. Prior to cystoscopic diagnostics (“gold standard“ for specificity), diagnostics included the qualitative UBC^®^ Rapid POC evaluation in comparison to the quantitative determination by UBC^®^ Rapid including the Concile^®^ Ω100 Reader, which measured the intensity of the colored line of the POC cassette.

For data evaluation, different thresholds of band intensity (cut-off values) for considering a test positive were applied for the determination of false positive or false negative values and the corresponding sensitivities, specificities, and positive and negative predictive values (PPVs and NPVs).

### 2.9. Tests

1. The UBC^®^ Rapid biomarker test for visual qualitative analysis: The UBC^®^ Rapid test bis a commercially available visual point-of-care (POC) test, detecting antigen fragments of cytokeratin 8 and 18 from urine samples qualitatively (arocell, Stockholm, Sweden). The UBC^®^ Rapid test uses qualitative immuno-chromatographic lateral flow assays, which develop a concentration-dependent color reaction as a cut-off value on a test line at a predefined concentration. A positive reaction is determined by the subjective decision of human operators.
2. The UBC^®^ Rapid Concile^®^ Ω100 photometric reader is a photometric reader for quantitative determination. Photometric readers represent a development including the objective, quantitative evaluation of POC assays. This reader is a commercially available measuring device for immediate patient diagnostics and delivers measurement results within 10 to 20 min (Concile GmbH, Freiburg, Germany). The lowest concentration detection level of the UBC^®^ Rapid Concile^®^ Ω100 photometric reader is 0.5 µg/L. For this reason, the resultant distribution curves in this investigation do not include concentration values below the detection limit of the quantitative POC determinations.
3. Urinstix (leukocyturia, hematuria, nitrite), which indicates a possible urinary tract infection.
4. Possibly other examinations, depending on the practice routine, e.g., urine cytology, ultrasound, etc.

## 3. Results

**Figure 1:**
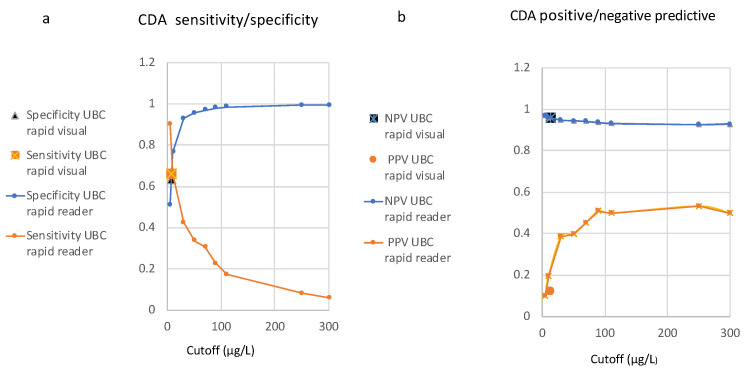

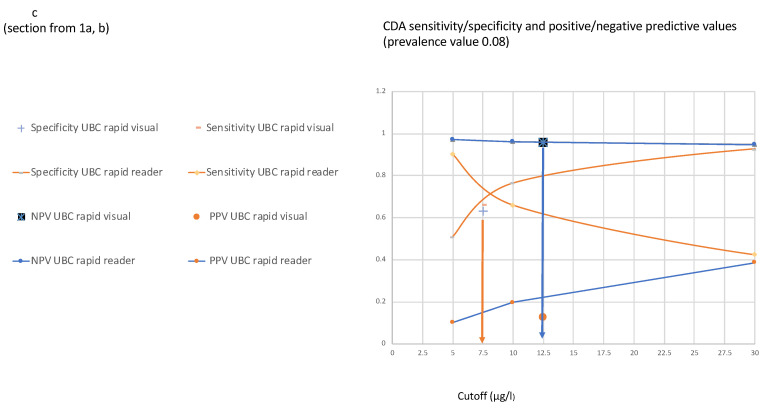
(**a**–**c**): Cumulative distribution analysis (CDA): Distribution of sensitivity and specificity (**a**) as well as positive and negative predictive values (**b**) on the y-axis along cut-offs (x-axis) of the UBC^®^ POC Rapid assay, determined using the Concile Ω100 reader, utilizing the study data at a prevalence value of 0.073. The cut-off concentrations for the qualitative test were derived graphically, as demonstrated in (**c**) (section of (**a**,**b**)).

CAD was employed for sensitivity/specificity (SS) and PPV/NPV analysis against the selected cut-offs from 0.5 to 300 µg/L, as shown in Figure 1a,b. The ratio between confirmed urinary bladder cancer (*n* = 91) and no evidence of urinary bladder cancer (*n* = 1152) reflects a prevalence value of 0.073 in the current study set. The respective curves for SS and PV values run in opposite directions along the cut-off distributions.

A section of both curves combined in a single graph (Figure 1c) was used to graphically determine the SS/PV cut-off concentrations of the visual test. The cut-off level of the qualitative assay was 7.5 µg/L, with a sensitivity of 65.9% and a specificity of 63.3%, and the PPV was 12.4% and the NPV was 95.9%, at a threshold value of 12.5 µg/L. 

**Figure 2:**
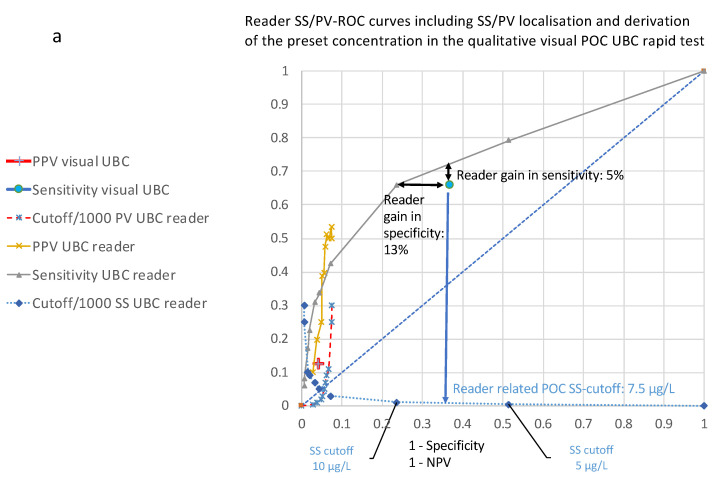

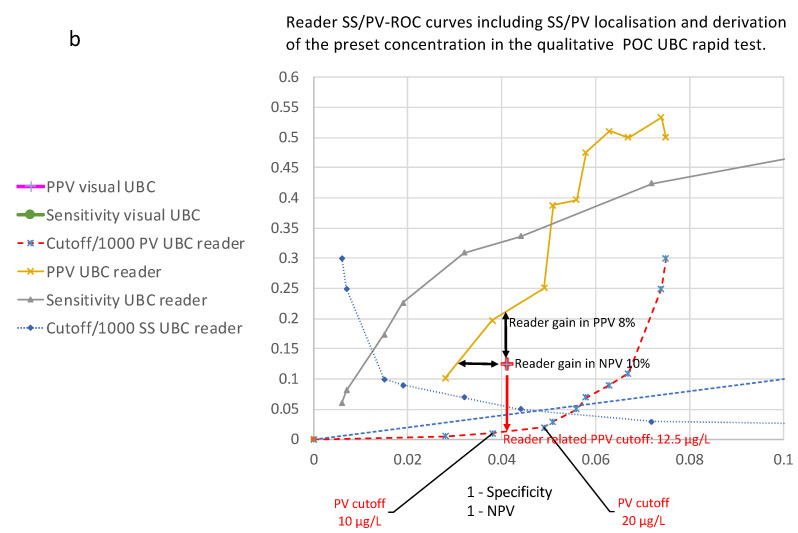
(**a**,**b**): Receiver operating characteristic (ROC) curves for the profile of sensitivity/specificity (SS) as well as for positive (PPVs) and negative predictive values (NPVs) along the cut-offs between 0 and 300 µg/L (all y-axis), including the graphically derived quantitative sensitivity/specificity (SS) and preset cut-off determination for the line in the qualitative visual test cassette, based on a comparison of their corresponding SS locations. (**b**): Section of (**a**), based on a comparison of their corresponding PPV/1-NPV locations.

For the graphic concentration determination of the qualitative test in ROC curves, a diagram was constructed including both the SS– and PV–ROC curves (SS/PV–ROC curve plot), including all of their respective cut-off values for the quantitative UBC^®^ Rapid test and the SS/PV values determined in the quantitative determination by the UBC^®^ Rapid test (Figure 2a).

The graph of the SS–ROC curve illustrates the profile of sensitivity/specificity and PPVs/NPVs for the whole range of preset cut-offs.

Qualitatively, within the shape of the SS–ROC curve, the risk score threshold increases above the diagonal along the ROC curve from the right-hand corner at the top of the plot to the bottom left-hand corner, and it has an AUC located above the diagonal. The PV risk score threshold increases in the opposite direction, from the left-hand corner to the bottom of the right-hand side.

At a given cut-off, the resultant values for sensitivity and PPV result in different false positive and true positive rates.

According to the results for sensitivity/specificity and the positive/negative predictive values, as well as the ROC curve distributions and their corresponding cut-off values within the same diagram, the preset unknown concentrations of the test line in the qualitative POC cassette were derived quantitatively for sensitivity/specificity graphically. Like in Figure 1c, the concentration for SS was found to be 7.5 µg/L (Figure 2a), and for predictive values, it was found to be 12.5 µg/L (Figure 2b). Based on these set concentrations, the reader-based evaluation shows a 5% increase in sensitivity and a 13% increase in specificity (Figure 2a) as compared to the visual (subjective determined) qualitative POC test. In the case of predictive values, there was a gain of 8% for the PPV and 10% for the NPV (Figure 2b).

**Figure 3:**
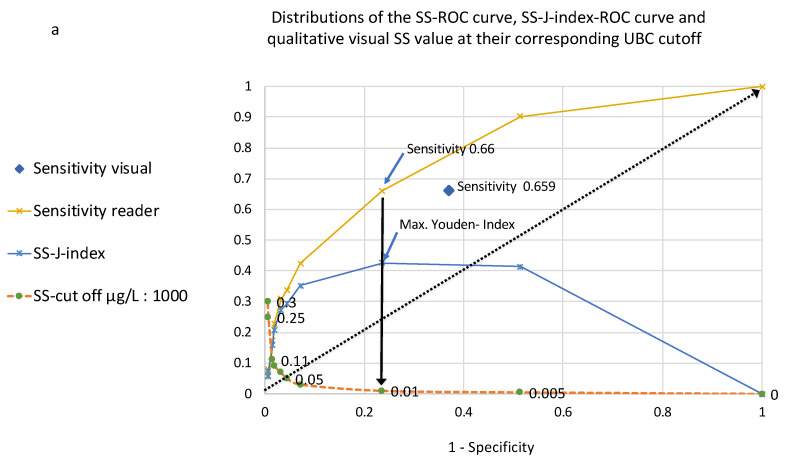

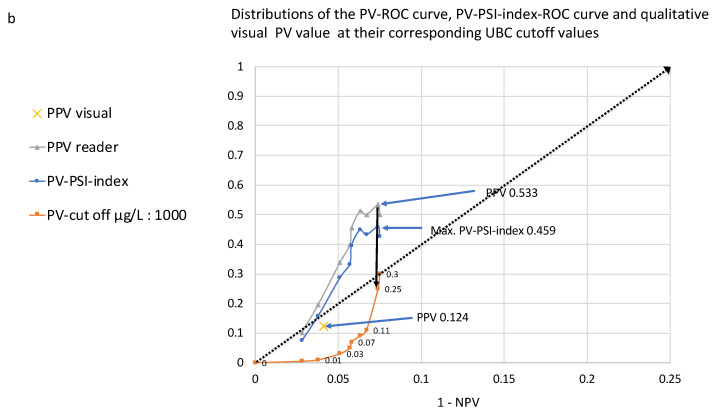
(**a**,**b**): Distributions of the SS–ROC curve, SS-J index–ROC curve, and qualitative visual SS (y-axis) at their corresponding UBC cut-off values at 1-specificity (x-axis). (**b**): Distributions of the PV–ROC curve, PV-PSI index–ROC curve, and qualitative visual PV at their corresponding UBC cut-off values (y-axis) in relation to 1-NPV (x-axis).

Based on the findings in Figure 1 and Figure 2, the SS-J/PV-PSI index values were calculated for all of their corresponding cut-off concentration values and index–ROC curves, with integrated cut-off value distribution curves constructed for SS and PV (Figure 3a,b).

In agreement with the SS–ROC curve, the maximal reader-related SS-J index was 0.425 at a cut-off of 10 µg/L, with a sensitivity of 0.66 and a specificity of 0.765 (Figure 3a). Additionally, in agreement with the PV–ROC curve, the maximal PV-PSI index was 0.459 at a cut-off of 250 µg/L, with a PPV of 0.5 and an NPV of 0.928 (Figure 3b), respectively.

**Figure 4:**
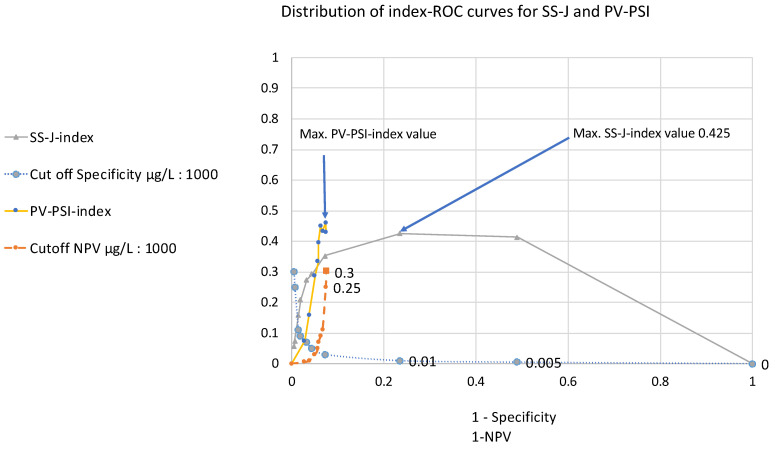
Distribution of index–ROC curves for SS-J and PV-PSI at their corresponding cut-off values (all y-axis) in the UBC assay compared to their corresponding values of 1-specificity and 1-NPV (x-axis).

Contrary to the SS-J index–ROC curves, the PV-PSI index–ROC curves (prevalence value 0.73) were only distributed within the range of the X-axis (1-NPV) from 0.028 to 0.075, whereas the SS–ROC curves fell within the range (1 − specificity) from 0 to 1 (Figure 4). This included a lack of AOX within the 0–1 range for 1-NPV (Figure 4).

A direct comparison of the two index curves in Figure 4 is complicated, because the cut-off levels run in opposite directions, as was already observed in the case of the SS/PV–ROC curves (Figure 1 and Figure 2).

**Figure 5:**
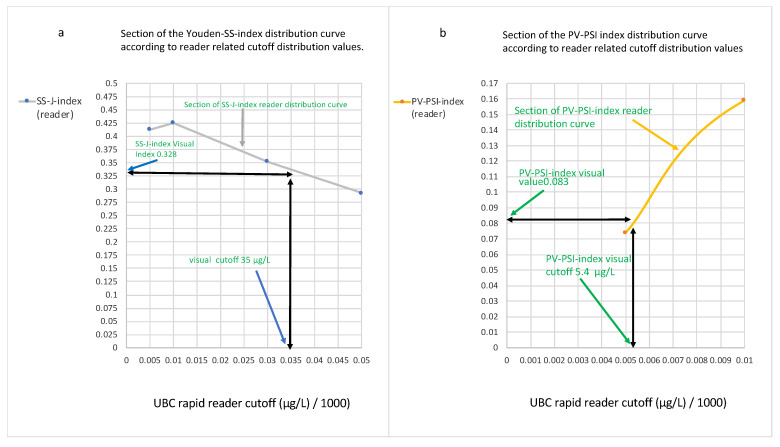

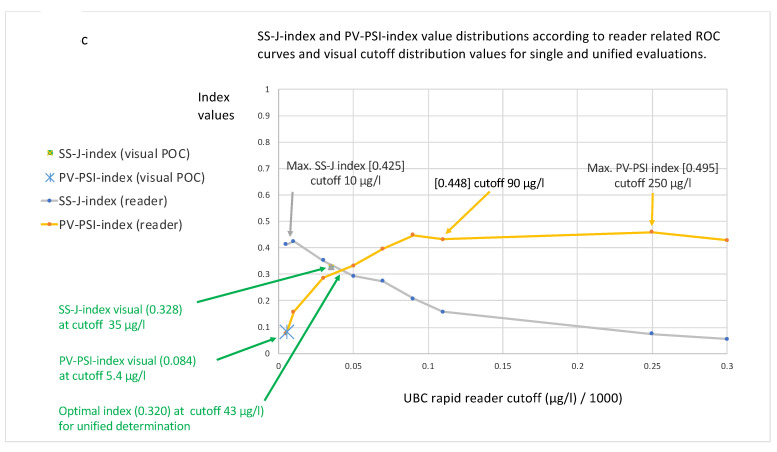

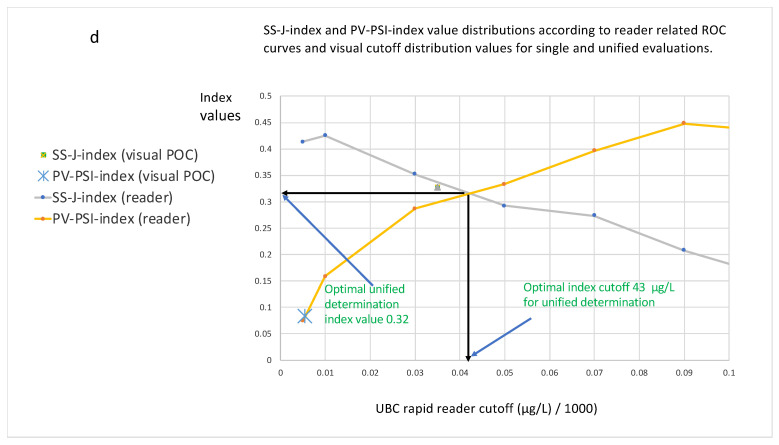
(**a**–**d**): Sections from SS-J index/PV-PSI index reader cut-off distribution curves. (**a**) Determination of visual SS-J index (y-axis) concentration values (cut-off 35 µg/L). (**b**) Derivation of visual PV-PSI index (y-axis) concentration values (cut-off 5.4 µg/L). (**c**): SS-J/PV-PSI index cut-off diagram: SS-J index, PV-PSI index reader, and visual POC test distributions (all y-axis) according to their cut-off concentration values, including maximal unified SS/PV index cut-off. (**d**): Section of (**c**). Quantitative estimation determination of maximal unified SS/PV index value (0.32) and the corresponding cut-off value (43 µg/L).

However, if the values are plotted in an SS-J/PV-PSI index cut-off diagram using the cut-off value on the X-axis, and the Y-axis is used for the indices, a direct comparison of both the reader index curves and the visual indices can be undertaken. In order to include the visual SS-J/PV-PSI index determinations, their respective concentrations were graphically derived using their indices from sections of the reader index cut-off distribution curves (Figure 5a,b), and the resulting values were integrated into the SS-J/PV-PSI index cut-off diagram (Figure 5c).

Figure 5c demonstrates the distributions of the SS-J index and PV-PSI index reader concentrations, as well as the visual POC test line SS-J and PV-PSI index concentrations according to their cut-off values in an SS-J/PV-PSI index cut-off diagram.

The cut-off value at which both curves cross within the diagram (43 µg/L) reflects the maximal index value (0.33) for unified SS-J and PV-PSI cut-off determination (Figure 5d). The visual POC SS-J index (0.328) at a cut-off of 35 µg/L is close to this area, whereas the visual POC PV-PSI index (0.083) at a cut-off of 5.4 µg/L is far away.

**Figure 6:**
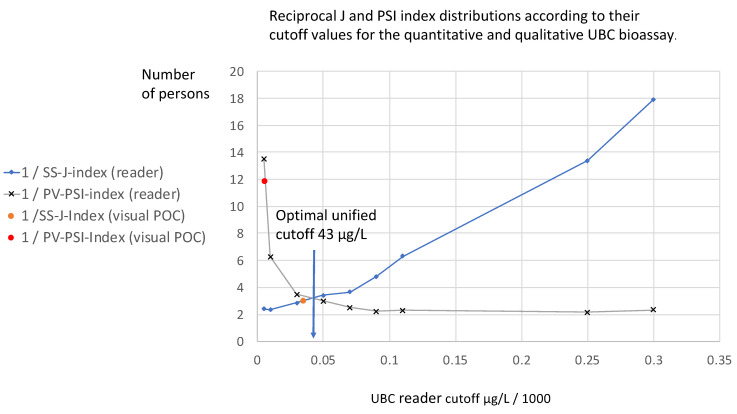
Reciprocal J and PSI index distributions (y-axis) according to their cut-off values (x-axis) for the quantitative and qualitative UBC^®^ Rapid test. These indicate the number of persons (y-axis) with a given disease who need to be examined in order to correctly detect one person with the disease (1/SS-J index), and the number of persons who need to be examined in order to correctly predict a diagnosis of the disease (1/PV-PSI index). The optimal unified cut-off (42.5 µg/L at 3.25 persons) is located at the intersection of both curves.

Figure 6 demonstrates the distributions of the reciprocal SS-J index and PV-PSI index reader concentrations, as well as the reciprocal visual POC test line SS-J and PV-PSI index concentrations according to their cut-off values in an SS-J/PV-PSI index cut-off diagram.

The cut-off value at which both curves cross within the diagram (43 µg/L) reflects the unified optimal diagnostic reciprocal index value (3.2 persons) for unified reciprocal SS-J and PV-PSI cut-off determination. The reciprocal visual POC SS-J index (0.328) at a cut-off of 35 µg/L is close to this area, whereas the reciprocal visual POC PV-PSI index (12 persons) at a cut-off of 5.4 µg/L is far away.

At increasing threshold distributions, the curves for the reciprocal J and PSI index distributions show inverse proportionality and have different shapes. Within the shape of the reciprocal values SS-J index curve, the number of persons increases gradually at increasing cut-off values, in contrast with the reciprocal PV-PSI index curve, which shows a sharp decrease similar to a half-life curve within the range of 5 µg/L to 90 µg/L, and continues at a constant level close to a value of two persons. In both curves, the qualitative visual POC test results are located very close their corresponding curves. 

## 4. Discussion and Conclusions

The effect of the threshold for test positivity on test sensitivity and specificity has been studied extensively in SS–ROC curve analysis, while there are as yet only very few publications concerning PV–ROC curves [23,24,25]. SS–ROC curves, as well as PV–ROC curves, include a summary of information, and some information is lost, particularly the actual value of the cut-off. For this reason, it has been necessary, until now, to supplement ROC curves with a cumulative distribution analysis (CDA), which displays the sensitivity and specificity against the cut-off values on the X-axis [26]. That is why this manuscript includes CDA as a traditional method and also uses it for the derivation of quantitative values (µg/L) from the qualitatively determined SS and PV values (Figure 1a–c).

Using the novel ROC curves developed with integrated cut-off distributions published within this manuscript, no information is lost and the derivation of concentrations for the qualitative visual results can be undertaken as well (Figure 2a,b). This means that for such investigations, CAD is not necessarily needed any more, and evaluations containing ROC curves with integrated threshold value distribution curves contain more useful information than those without.

It is already known that qualitatively, within the shape of the SS–ROC curve, the risk score threshold increases above the diagonal along the ROC curve from the right-hand corner at the top of the plot to the bottom left-hand corner, and the PV risk score threshold increases in the opposite direction, from the top left-hand corner to the bottom of the right-hand side [23,24,25]. However, to the best of my knowledge of the present literature, no other author has ever published SS– or PV–ROC curves with integrated cut-off distribution curves (Figure 2, Figure 3 and Figure 4).

Using these new approaches and plotting both SS–ROC curves and PV–ROC curves in a single diagram (e.g., Figure 2), it can be directly demonstrated that the threshold distributions run in opposite directions. At a given cut-off, the resultant values for sensitivity and PPV are seen at different false positive and true positive rates, and in the case of the SS-J index– and PV-PSI index–ROC curves (Figure 4), the threshold distributions run in opposite directions as well.

Another advantage is that data from the visual POC single-point SS or PV evaluation can directly be compared with the reader evaluation in a single graph by comparing their locations on the X- and Y-axes to the corresponding points in the ROC curves.

One of the goals in our clinical investigation was to find out whether the introduction of quantitative readers for POC line evaluations can improve the clinical performance of POC assays. This could be calculated for SS and PV determinations within a single diagram. The reader-related method added diagnostic gains both in sensitivity/specificity (5% and 13%, respectively, Figure 2a) and PPVs/NPVs (8% and 10%, respectively, Figure 2b). Such a compact, easy, and informative unified analysis using a single diagram is a novel approach, which has so far never been published in the literature.

The indices described in this manuscript are a summation of the effectiveness of SS and PPVs/NPVs. In their publication on PSI, Linn and Grunau wrote “There is a vast literature on the use of J in assessing the ROC in continuous diagnostic tests. Similarly, future studies should explore PSI for continuous diagnostic tests” [27]. According to the present literature, the SS-J/PV-PSI index–ROC curves presented in this manuscript represent the first effort at integrating their proposal.

In Figure 3a,b, the ROC curves for SS (Figure 3a) and PV (Figure 3b) are compared to their corresponding index–ROC curves. The SS–ROC curve has an AUC, the SS-J index–ROC curve has an area over the X-axis (AOX), and they have a theoretical range from 0 to 1, whereas this is not the case for the PV–ROC curve and the PV-PSI index–ROC curve, which cover only a restricted area over the X-axis. It is noteworthy that the shape of the PV–ROC curve changes substantially when the prevalence shifts from >1 to <1 [23,24,25]. This is why it does not make much sense to compare PV values for the same biomarker in different clinical studies including different prevalence values. 

In Figure 3a, the sensitivity of the visual determination is equal to the maximum SS-J index-associated reader sensitivity, but the specificity is lower, and for this reason, the diagnostic reader test system provides a better result. In Figure 3b, the PPV (0.124) of the visual determination is 40% lower, and even although the NPV is high (0.959), the visual test is inferior to the reader index-determined maximal PV value, and for these reasons, the reader test system has the better overall clinical performance. 

The SS-J index– and PV-PSI index–ROC curves and the locations of the maximal index values in Figure 4 differ in their distributions. Youden index–ROC curves, which are not affected by prevalence, always show a result from 0 to 1 over the vertical axis, but their form over the axis can change according to the sensitivity and specificity of a bioassay. The shape of PV-PSI index curves can change due to the PPV and NPV as well, but in addition, they can move according to the values of prevalence, e.g., from the left side of the graph to the right at increasing prevalence [23,24]. Because this investigation had a very low prevalence value (0.073), the curve is located on the very left side of the graph. It starts at a cut-off value of 5 µg/L and ends at a cut-off value of 250 µg/L at a 1-NPV of 0.072. However, at a cut-off value of 300 µg/L, this result was not theoretically expected. The explanation for this is a problem already known for such predictive value clinical bioassay evaluations [25]: at increasing concentration levels, the number of true positive (TP) and false positive (FP) test results decreases in the calculations for PPV, even when the study includes a high number of patients. In this study, the values for TP and FP were eight and seven at a cut-off of 250 µg/L, and the value for both was six at a cut-off of 300 µg/L. Due to these statistical conditions, the expected values did not end at a 1-NPV of 0.073, as the mathematically expected level, and the curve deviated from its direction for the last two index values. 

A direct comparison of the two index curves in Figure 4 turned out to be complicated, because the cut-off levels run in opposite directions. This problem increases when cut-off distributions, sensitivity, and PV are plotted against specificity and 1-NPV for two different assay systems, as was observed in the case of the *SS*/PV- and SS-J/PV-PSI index–ROC curves (Figure 2 and Figure 3).

One of the goals of this study was to directly evaluate and compare the quantitative and qualitative bioassays according to SS and PV values; another goal was to show how SS and PV results differ in their clinical applicability and whether a method can be proposed as a transparent means to identify appropriate cut-offs for both sensitivity/specificity and predictivity at a given cut-off level.

Because the unified comparative evaluation of SS– and PV–ROC curves cannot be performed using the value of the AUC (PV- and PV-PSI index–ROC curves have no AUC), it was also desirable to find an alternative approach to overcome this limitation.

By using indices, data for sensitivity, specificity, and predictive values were normalized, because the indices use only numbers independent of designations like %, ng/mL, or units. In an SS-J/PV-PSI index cut-off diagram, by plotting the cut-off value on the X-axis and the indices on the Y-axis, any SS-J index value can be directly related to its corresponding PV/PSI index values at varied cut-off levels.

As demonstrated in Figure 5, this new tool is useful for the simultaneous comparison and selection of unified or separate optimal cut-off values, or to find out whether a common cut-off level can be applied according to clinical questions like diagnosis, follow-up, or screening. A maximal Youden or PSI index can be regarded to be the optimal choice of cut-off, provided it can be assumed that the sensitivity and specificity or PPV and NPV are of equal diagnostic importance. Concerning the visual indices, this method clearly demonstrates that the visual POC PV-PSI index (0.083) at a cut-off of 5.4 µg/L is far away from the optimal unified SS-J/PV-PSI index cut-off. This means that a single preset cut-off line on the test cassette does not simultaneously include a valuable diagnostic result for the detection and prediction of cancer in the study group and the visual test line cannot be recommended for unified determination.

Other considerations for the methods described in this manuscript, however, depending on the aim of an investigation, may prevail. The SS-J/PV-PSI index cut-off diagram includes the corresponding indices for cut-off levels within the complete range of an assay and is, therefore, useful for special clinical considerations that require the clinical diagnostic parameters mentioned to vary.

Because the curves within an SS-J/PV-PSI index cut-off diagram are distributed over the complete cut-off range of a quantitative assay, even more applications exist. Complete or partial areas over the X-axis can be calculated for summarized quantitative effectivity evaluations, with respect to single and/or unified SS-J and PV-PSI indices and with respect to single, separate, or several unified cut-off thresholds. The SS-J/PV-PSI index cut-off diagram solves the mentioned problem for the comparison of SS– and PV–ROC curves according to the AOC area.

As shown in Figure 6, with respect to the complete concentration range of the UBC^®^ Rapid POC test, a correct detection or prediction based on fewer than two persons cannot be achieved. This means that the test cannot be used for screening in the investigated population.

While the reciprocal SS-J index of the qualitative test can detect one out of three patients, the reciprocal PV-PSI index gives only one out of twelve patients a correct diagnosis. Accordingly, the quantitative test determination only provides valuable information on SS. Alternatively, the quantitative reader evaluation allows us to select separate cut-off values; for example, an SS threshold of 10 µg/L is needed to correctly detect 1 person out of 2.3 patients with the disease, while a PV threshold of 90 µg/L is required to correctly predict a diagnosis in 1 out of 2.2 patients. The optimal unified cut-off (42.5 µg/L at 3.25 persons) leads to an approximately 50% additional increase, which seems not to be recommendable.

In contrast to SS–ROC curves, the distribution of PV–ROC curves depends on prevalence. Concerning Figure 5c and Figure 6, a general judgement of the results for predictive values can only be made with regard to the (low) prevalence value (0.073) in the study. It is known from mathematical evaluations that PPV values increase with increasing prevalence, while NPV values decrease [23,24]. However, the data in this study and the prevalence reflect the daily routine of urological practice facilities and can be regarded to be valuable information for the practicing urologist.

Both the SS-J/PV-PSI index cut-off diagram and the diagram using their reciprocal indices include new technical and clinical diagnostic tools, which might also be used in a broad range of applications like plant epidemiology [18,19], in the evaluation of machine learning algorithms [20], or to improve the accuracy, scalability, and performance of graph neural networks [21].

## Data Availability

The documents containing the clinical data are held by the author and are stored in compliance with data protection regulations.
